# Autoantibodies against Muscarinic Receptors in Breast Cancer: Their Role in Tumor Angiogenesis

**DOI:** 10.1371/journal.pone.0057572

**Published:** 2013-02-27

**Authors:** María Gabriela Lombardi, María Pía Negroni, Laura Tatiana Pelegrina, María Ester Castro, Gabriel L. Fiszman, María Eugenia Azar, Carlos Cresta Morgado, María Elena Sales

**Affiliations:** 1 Laboratorio de Inmunofarmacología Tumoral, Centro de Estudios Farmacológicos y Botánicos (CEFYBO)-CONICET, UBA. Buenos Aires, Argentina; 2 Pathology Department, Medical School, University of Massachusetts, Worcester, Massachusetts, United States of America; 3 Instituto de Oncología A.H. Roffo, UBA, Buenos Aires, Argentina; University of Michigan School of Medicine, United States of America

## Abstract

The presence of autoantibodies in cancer has become relevant in recent years. We demonstrated that autoantibodies purified from the sera of breast cancer patients activate muscarinic acetylcholine receptors in tumor cells. Immunoglobulin G (IgG) from breast cancer patients in T1N0Mx stage (tumor size≤2 cm, without lymph node metastasis) mimics the action of the muscarinic agonist carbachol stimulating MCF-7 cell proliferation, migration and invasion. Angiogenesis is a central step in tumor progression because it promotes tumor invasion and metastatic spread. Vascular endothelial growth factor-A (VEGF-A) is the main angiogenic mediator, and its levels have been correlated with poor prognosis in cancer. The aim of the present work was to investigate the effect of T1N0Mx-IgG on the expression of VEGF-A, and the *in vivo* neovascular response triggered by MCF-7 cells, via muscarinic receptor activation. We demonstrated that T1N0Mx-IgG (10^−8^ M) and carbachol (10^−9^ M) increased the constitutive expression of VEGF-A in tumor cells, effect that was reverted by the muscarinic antagonist atropine. We also observed that T1N0Mx-IgG and carbachol enhanced the neovascular response produced by MCF-7 cells in the skin of NUDE mice. The action of IgG or carbachol was reduced in the presence of atropine. In conclusion, T1N0Mx-IgG and carbachol may promote VEGF-A production and neovascularization induced by breast tumor cells *via* muscarinic receptors activation. These effects may be accelerating breast tumor progression.

## Introduction

The presence of autoantibodies (autoAbs) against tumor associated antigens in the sera of cancer patients has been previously reported [1]. Moreover, several studies reviewed by Fernández Madrid et al. [1] indicate that a plethora of autoAbs with different specificities has been found in breast cancer patients. These spontaneous responses are frequently detected in 5 to 30% of patients for one autoantigen. Chapman et al. [2] reported data from breast carcinoma patients that confirmed that autoAbs to tumor associated antigens can be measured up to four years before mammography imaged the tumor. Their results also strengthen previous ones, since they reported that the incidence of autoAbs, to at least one of six tumor associated antigens (p53, c-myc, HER2, NYESO-1, BRCA2, and MUC1) analyzed as a group rises to 64% in the sera of breast carcinoma patients. These striking data imply that the human immune system detects the tumor antigens as “nonself” and makes a humoral immune response very early in the disease process. Even though the presence of autoAbs has been extensively analyzed by sensible methods, the function of these autoAbs during tumor progression has not been fully understood yet. Emerging evidence indicates that most of the antigens identified in human tumors are self-proteins without mutations but inappropriately expressed or over-expressed [1].

Muscarinic acetylcholine receptoŕs (mAChR) expression is up-regulated in different types of tumors such as colon, lung, ovarian and prostate tumors [3]. These receptors are part of the G-protein-coupled receptors family, there are five different subtypes of them (M_1_–M_5_), and they bind acetylcholine (ACh) [4]. Previous work in our laboratory demonstrates that mAChR are over-expressed in tissue from human breast cancer tumors in comparison to breast normal tissue [5]. Moreover, IgG from patients with breast cancer in early stages can promote tumor proliferation due to the activation of mAChR [6]. Hence, autoAbs against mAChR could be playing an important role in tumor growth.

One of the most important steps in tumor progression is angiogenesis, the process that leads to tumor neovascularization by new blood vessel formation to promote tumor growth and metastatic spread [7]. The development of new capillaries is regulated by a complex mechanism with the participation of pro-angiogenic factors. Among them is the vascular endothelial growth factor-A (VEGF-A), which stimulates endothelial cells survival, proliferation and migration allowing the invasion of the surrounding tissue, and the formation of blood vessels. These functions are triggered by the interaction of VEGF-A with its tyrosine kinase receptors, which in turn transmits signals to various downstream proteins [8]. Taking into account that previous studies indicate that autoAbs against mAChR could have a role in tumor development, and the relevance of angiogenesis in tumor growth, then we wanted to know if autoAbs against mAChR in breast cancer patients could influence tumor angiogenic response. In consequence, we investigated the role of autoAbs present in the immunoglobulin G (IgG) fraction of breast cancer patients in stage I on VEGF-A levels produced by MCF-7 cells and on tumor neovascular response induced in an *in vivo* model, focusing on the participation of mAChR. We demonstrated that IgG purified from the sera of breast cancer patients in stage I increased the constitutive expression of VEGF-A in tumor cells, effect that was reverted by the muscarinic antagonist atropine. We also observed that IgG enhanced the neovascular response produced by MCF-7 cells in the skin of NUDE mice. Both effects were similar to those produced by the cholinergic agonist carbachol.

## Materials and Methods

### Selection of Patients

Cancer patients with breast adenocarcinoma in stage I were selected in the Mastology Department of the Angel H. Roffo Oncology Institute, University of Buenos Aires, and were classified according to TNM criteria as T1N0Mx (tumor size≤2 cm, without axillary node metastasis). Subjects free of illness (normal) or with breast benign fibroadenoma (BFA) were used as controls. All of them were free of treatment, and blood samples (10–20 ml/donor) were obtained at routine procedures before surgery. Written informed consent was approved by the Ethics in Research Committee from the A.H. Roffo Oncology Institute and was obtained from each patient. This study was also approved by the Ethics Committee from the Centro de Estudios Farmacológicos y Botánicos (CEFYBO)-CONICET.

### IgG Purification

After centrifugation of blood samples, sera were separated and heat-inactivated at 56°C for 30 min. IgG purification was performed by affinity chromatography in protein G-agarose (Invitrogen Inc., Carlsbad, CA, USA). Samples (pH 6.0–7.5) were loaded onto the column after equilibrating it with 10 volumes of binding buffer: 0.01 sodium phosphate pH 7.0; 0.15 M NaCl at high salt concentration. Elution was performed with 0.1 M glycine hydrochloride (pH 2.6) and adjusted immediately to pH 7.0. Concentration of proteins in the collected fractions was obtained by measuring the absorbance at 280 nm, and fractions that corresponded to the peak of maximal absorbance were stored at −20°C.

### Cell Culture and Animals

The human breast adenocarcinoma cell line MCF-7 was obtained from the American Type Culture Collection (ATCC; Manassas, VI, USA), and cultured in Dulbecco’s modified Eagle’s medium and F12 medium (DMEM:F12; 1∶1; Invitrogen Inc., Carlsbad, CA, USA) with 2 mM L-glutamine, 80 µg/mL gentamycin, supplemented with 10% heat inactivated fetal bovine serum (FBS) (PAA laboratories GmbH, Haidmannweg, Austria) at 37°C in a humidified 5% CO_2_ air. MCF-10A cells were also purchased by ATCC and constitute a non tumorigenic cell line derived from human mammary tissue. Cells were grown on tissue culture plastic dishes, in DMEM:F12 medium supplemented with 10% FBS, hydrocortisone (0.5 µg/ml), insulin (10 µg/ml), and epidermal growth factor (20 ng/ml). The tumor cell line, LMM3, was previously obtained from the murine syngeneic mammary adenocarcinoma MM3 [9]. Cell cultures were maintained as monolayers at 37°C and 5% CO_2_ in DMEM medium supplemented with 5% FBS. All cell lines were detached using the following buffer: 0.25% trypsin and 0.02% EDTA in Ca^2+^ and Mg^2+^-free phosphate buffer saline (PBS) of confluent monolayers. The medium was replaced three times a week. Cell viability was assayed by Trypan blue exclusion test, and the absence of mycoplasma was confirmed by Hoechst staining [10].

Female NUDE mice or BALB/c mice, three months old, were purchased from the colony of the animal facility of the National Commission of Atomic Energy (CNEA, Buenos Aires, Argentina) that were kept under specific pathogen-free conditions following the protocol designed by the National Institute of Health (USA) in the Guidelines for the care and handling of laboratory animals (1986) and protocols were approved by the Internal Committee for the Care and Use of Laboratory Animals (CICUAL) from the School of Medicine, University of Buenos Aires.

### Western Blot Assays

MCF-7, MCF-10A or LMM3 cells (2×10^6^/well) were seeded in 6-well plates with 1 ml of DMEM:F-12 medium (Invitrogen Inc., Carlsbad, CA, USA) plus 10% FBS. Then, cells were treated with carbachol (10^−9^ M), or 10^−8^ M: T1N0Mx-, normal- or BFA-IgGs during 1 h in the absence or presence of 10^−8^ M muscarinic antagonists: atropine, 4-diphenylacetoxi-N-methylpiperidine (4-DAMP), or tropicamide that were added 30 min previous to carbachol. After treatment, medium was replaced by fresh medium plus 10% FBS. Supernatants from MCF-7, MCF-10A or LMM3 cell cultures were collected after 24 h and stored at −80°C. Cells were washed twice with PBS and lysed in 1 ml of modified RIPA buffer: 50 mM Tris–HCl pH 7.4, 50 mM NaCl, 5 mM NaF, 5 mM MgCl_2_, 1 mM EDTA, 1 mM EGTA, 5 mM PMSF, 1% Triton X-100, 10 µg/ml trypsin inhibitor, 10 µg/ml aprotinin, and 10 µg/ml leupeptin. After 1 h in ice bath, lysates were centrifuged at 8,000×g for 20 min at 4°C. Resulting supernatants were stored at −80°C, and protein concentration was determined by the method of Bradford [11].

Cell lysates or supernatants (80 µg protein/lane) were subjected to 12% sodium dodecyl sulfate-polyacrylamide minigels electrophoresis (SDS-PAGE). Then, proteins were transferred to nitrocellulose membranes (Bio Rad) and were blocked in 20 mM Tris-HCl buffer, 500 mM NaCl, and 0.05% Tween 20 (TBS-T) with 5% skimmed milk for 1 h at 20°C to 25°C. The nitrocellulose strips were subsequently incubated overnight with a specific rabbit anti-VEGF-A antibody diluted 1∶100 (Santa Cruz Biotechnology, Inc. Santa Cruz, CA, USA) in TBS-T. After several rinses with TBS-T, strips were incubated with the second antibody: goat anti-rabbit IgG conjugated with horseradish peroxidase (HRP) diluted 1∶20,000 (Sigma Chemical Co. St. Louis, MO, USA) in TBS-T with 3% skimmed milk at 37°C during 1 h. Bands were detected by enhanced chemiluminescence (ECL) [12]. Glyceraldehyde 3-phosphate dehydrogenase (GAPDH) was used as loading control. Quantification of the bands was performed by densitometric analysis using Image J program (NIH) and was expressed in optical density (O.D.) units relative to GAPDH.

### Tumor Induced Angiogenesis

Tumor induced angiogenesis was quantified with an in vivo bioassay previously described [13]. Briefly, tumor cell suspensions were prepared by detaching and washing MCF-7, MCF-10A or LMM3 cells twice with culture medium. Cell concentration was adjusted to 2×10^6^ cells/ml in: DMEM:F-12 medium for MCF-7 and MCF-10A cells, or in DMEM medium for LMM3 cells. Female NUDE mice were inoculated intradermically (i.d.) in both flanks with 0.1 ml of MCF-7 or MCF-10A cell suspension. BALB/c mice were also inoculated i.d. in both flanks with 0.1 ml of LMM3 cell suspension. Before inoculation MCF-7 and LMM3 cells were treated during 1 h with 10^−8^ M T1N0Mx-IgG or 10^−9^ M carbachol in the absence or presence of 10^−8^ M muscarinic antagonists: atropine, 4-DAMP or tropicamide, added 30 min previous to IgG or carbachol. Normal-IgG or BFA-IgG were used as control. Cells were washed before inoculation. After 5 days, the animals were sacrificed and skin removed by dissection of adjacent tissues. The vascular response was observed in the inner surface of the skin with a dissecting microscope (Konus USA Corporation, Miami, FL) at a 6.4× magnification, and the sites of inoculation were photographed with an incorporated digital camera (Canon Power Shot A75, Canon USA., Inc. Lake Success, NY). Photographs were projected onto a reticular screen to count the number of vessels per mm^2^ of skin. Angiogenesis was quantified as vessel density (δ), determined by the formula: Σ number of vessels in each square/total number of squares. Since MCF-7 tumor cells growth is estrogen dependent, animals were treated with β-estradiol injected subcutaneously (0.1 µg/mice dissolved in 0.1 ml ethanol: water) five days before the administration of tumor cells [14].

### Drugs

All drugs were purchased from Sigma Chemical Co (St. Louis, MI, USA) unless otherwise stated. Solutions were prepared fresh daily.

### Statistics

Results are given as means±S.E.M. of at least three independent experiments. The statistical significance of differences between means were analyzed by the analysis of variance using GraphPad Prism 5; *p*<0.05 was considered to be statistically significant. The analysis was complemented by using the Tukey test to compare among mean values. Differences between means were considered significant if *p*<0.05.

## Results

### Effect of Breast Cancer Patients’ IgG or Carbachol on VEGF-A Expression in Tumor Cells. mAChR Participation

**Figure 1:**
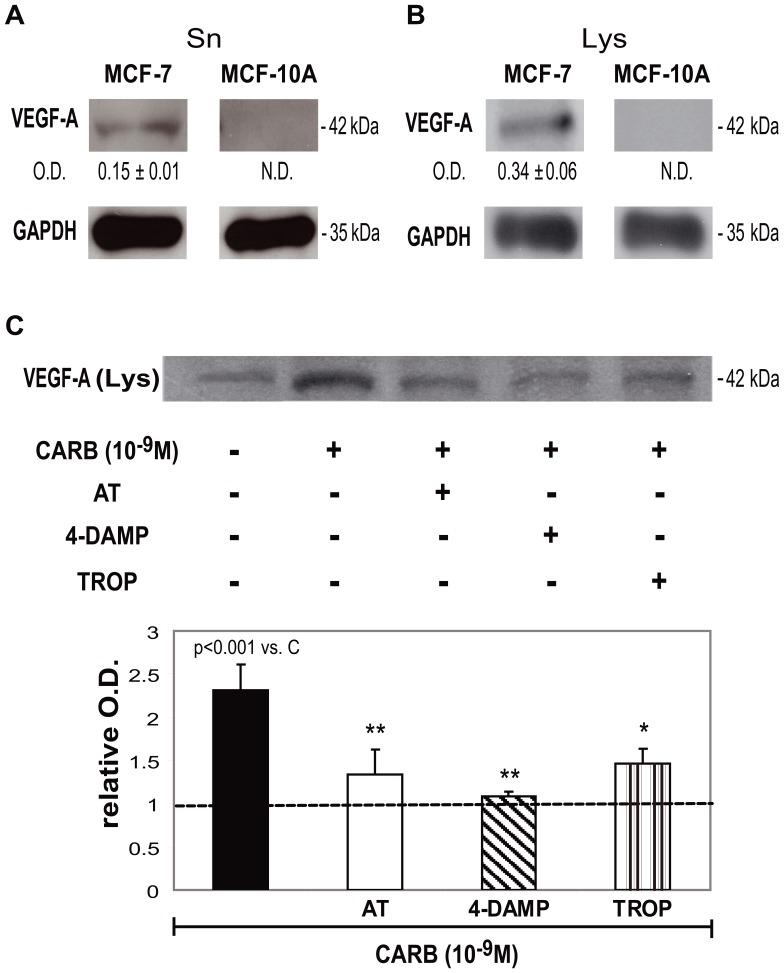
Effect of carbachol on VEGF-A expression in MCF-7 cells. Western blot assays were performed in A) supernatants (Sn) and B) lysates (Lys) from MCF-7 and MCF-10A cells. Optical density (O.D.) of the bands was calculated by densitometric analysis, and values were relativized to the expression of glyceraldehyde-3-phosphate dehydrogenase (GAPDH). N. D. not detectable. One representative experiment of three is shown. Values are mean ± S.E.M. of three experiments. Molecular weights are indicated on the right. C) MCF-7 cells were treated for 1 h with 10^−9^ M carbachol (CARB) in the absence or presence of 10^−8^ M atropine (AT), 4-diphenylacetoxi-N-methylpiperidine (4-DAMP), or tropicamide (TROP) and VEGF-A expression was measured in Lys. Densitometric analysis of the bands is shown in lower panel. Values were relativized to the expression of VEGF-A in MCF-7 cells without treatment, considered as 1, and is represented by a dotted line. Molecular weights are indicated on the right. Values are mean±S.E.M of three experiments. *p<0.1; **p<0.01 *vs*. CARB.

Since tumor cells usually express VEGF-A, we tested the presence of this factor in MCF-7 cell supernatants and lysates. We observed that tumor cells liberate VEGF-A into the cell culture supernatant (Fig. 1A) and that VEGF-A is present in cell lysates (Fig. 1B). We could not detect VEGF-A expression in normal MCF-10A cells either in culture supernatants or in cell lysates (Fig. 1A and B). Since we had previously demonstrated that MCF-7 cells express functional mAChR subtypes 3 and 4, we tested the action of carbachol on VEGF-A production by tumor cells. We observed that 10^−9^ M carbachol, significantly increased VEGF-A production by 133±30% in MCF-7 cells in comparison to control (cells without treatment, considered as 100%) (Fig. 1C). In addition, we observed that the action of carbachol was significantly reduced by: the non selective muscarinic antagonist atropine (42±12%), by the M_3_ receptor antagonist 4-DAMP (53±3%) or by tropicamide (37±8%) an M_4_ selective antagonist, confirming the participation of these receptors in carbachol-induced VEGF-A production by tumor cells.

**Figure 2:**
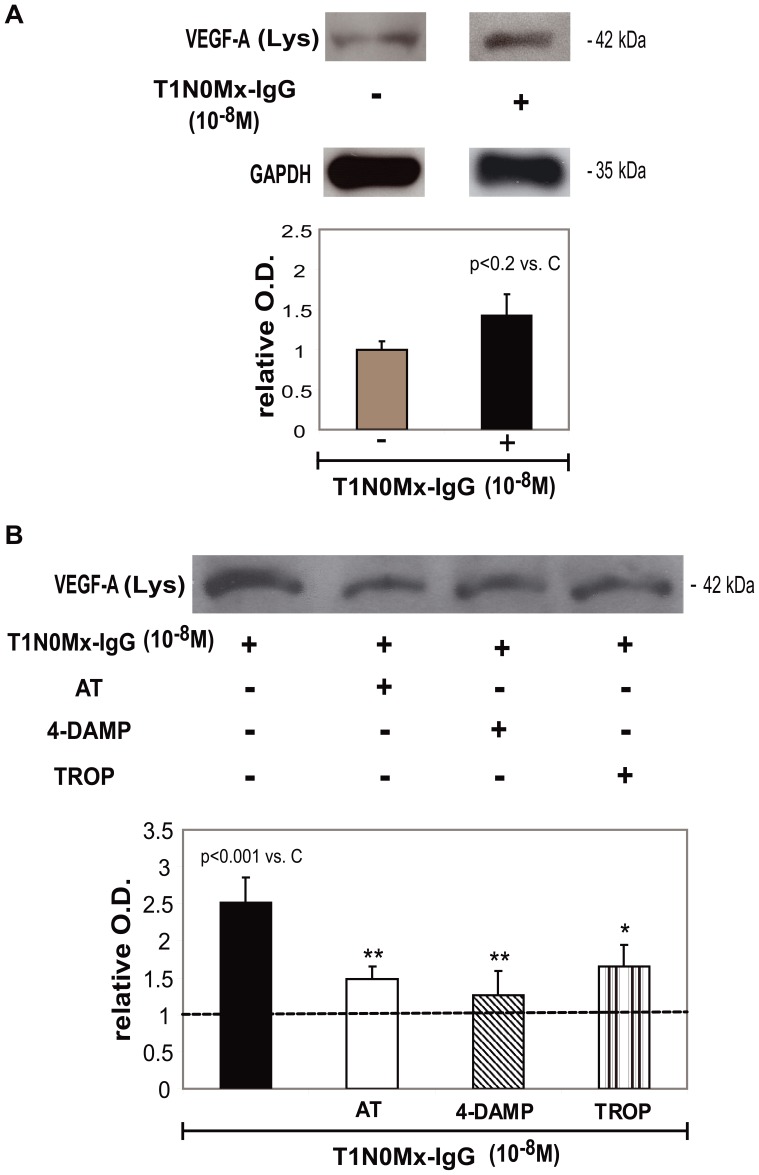
Effect of T1N0Mx-IgG on VEGF-A expression in MCF-7 cells. Western blot assays were performed in cell lysates (Lys) obtained from A) untreated MCF-7 cells or treated with IgG from breast cancer patients in stage I (T1N0Mx-IgG) (10^−8^ M) during 1 h in the absence or B) presence of 10^−8^ M atropine (AT) 4-diphenylacetoxi-N-methylpiperidine (4-DAMP), or tropicamide (TROP). Densitometric analysis of the bands is shown. Values were relativized to the expression of glyceraldehyde-3-phosphate dehydrogenase (GAPDH) and to the expression of VEGF-A in MCF-7 cells without treatment considered as 1, and is represented by a dotted line. Molecular weights are indicated on the right. Values are mean±S.E.M. of three experiments. *p<0.01; **p<0.001 *vs.*T1N0Mx-IgG.

**Figure 3:**
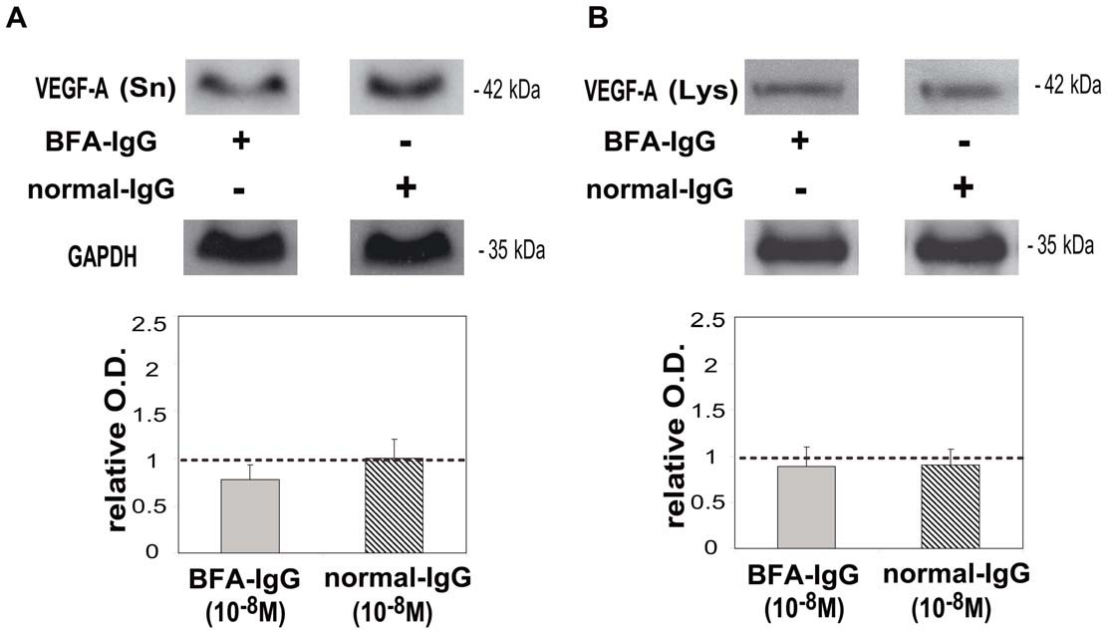
Effect of BFA-IgG and normal IgG on VEGF-A expression in MCF-7 cells. Tumor cells were treated with 10^−8^ M benign fibroadenoma (BFA) or normal-IgG during 1 h and Western blot assays were performed in MCF-7 cell A) supernatants (Sn) and B) lysates (Lys). Densitometric analysis of the bands is shown. Values were relativized to the expression of glyceraldehyde-3-phosphate dehydrogenase (GAPDH) and to VEGF-A in MCF-7 cells without treatment considered as 1, and is represented by a dotted line. Molecular weights are indicated on the right. Values are mean±S.E.M. of three experiments.

Then we investigated the action of T1N0Mx-IgG obtained from five different breast cancer patients in stage I, on VEGF-A expression in MCF-7 cells. The addition of breast cancer patients’ IgG increased VEGF-A liberation by tumor cells into the culture supernatants by 64±7% in comparison to control (MCF-7 cells without treatment) (Fig. 2A). We also demonstrated that T1N0Mx-IgG increased by 151±30% VEGF-A expression in tumor cell lysates in comparison to control (Fig. 2B). To analyze which subtype of mAChR was involved in the action of T1N0Mx-IgG we tested its action in the presence of muscarinic antagonists and we observed that atropine and 4-DAMP reduced IgG action by 41±6% and 50±12% respectively and in a more potent manner than tropicamide (34±10%) (Fig. 2C, lower panel). We also tested the action of normal-IgG or BFA-IgG (10^−8^ M) (n = 3), and we did not observe any effect on VEGF-A expression either in the supernatants or in the lysates obtained from tumor cells in comparison to control (MCF-7 cells without treatment, considered as 1) (Fig. 3A and B).

**Figure 4:**
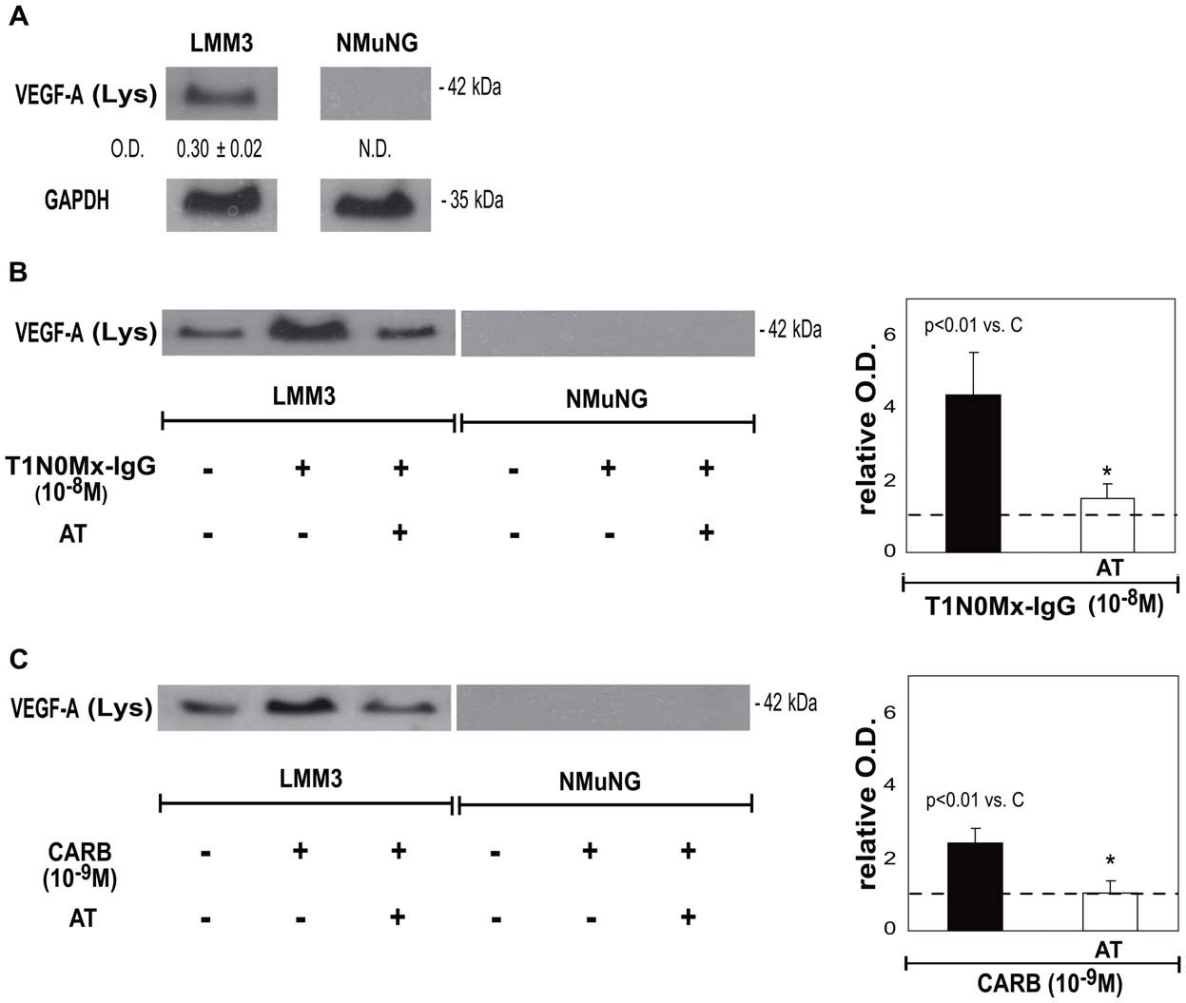
Effect of T1N0Mx-IgG on VEGF-A expression in murine tumor cells. A) LMM3 and NMuMG cell lysates (Lys). Optical density (O.D.) of the bands was calculated by densitometric analysis, and values were relativized to the expression of glyceraldehyde-3-phosphate dehydrogenase (GAPDH). N. D. not detectable. One representative experiment of three is shown. Values are mean±S.E.M. Molecular weights are indicated on the right. LMM3 cells were treated with B) T1N0Mx-IgG or C) carbachol (CARB) in the absence or presence of 10^−8^ M atropine (AT). Densitometric analysis of the bands is shown in the right pannel. Values were relativized to the expression of VEGF-A in LMM3 cells without treatment considered as 1, and is represented by a dotted line. Values are mean±S.E.M. of three experiments. *p<0.01 *vs.* T1N0Mx-IgG or CARB. Molecular weights are indicated on the right.

In addition, we analyzed the ability of T1N0Mx-IgG to cross react with mAChR present in LMM3 murine mammary tumor cells, and to modulate VEGF-A expression. As it is shown in Fig. 4A, LMM3 cells express this factor while normal murine mammary cells NMuMG, do not. The addition of 10^−8^ M T1N0Mx-IgG potently up-regulated VEGF-A expression more than 4 folds in cell lysates. This effect was significantly reduced by atropine, revealing mAChR participation in this effect (Fig. 4B). As a positive control, we evaluated the action of 10^−9^ M carbachol on murine tumor cells and it increased 1.5 fold VEGF-A protein expression in LMM3 cell lysates (Fig. 4C).

### Effect of IgG from Breast Cancer Patients and Carbachol on Tumor-induced Angiogenesis. mAChR Participation

**Figure 5:**
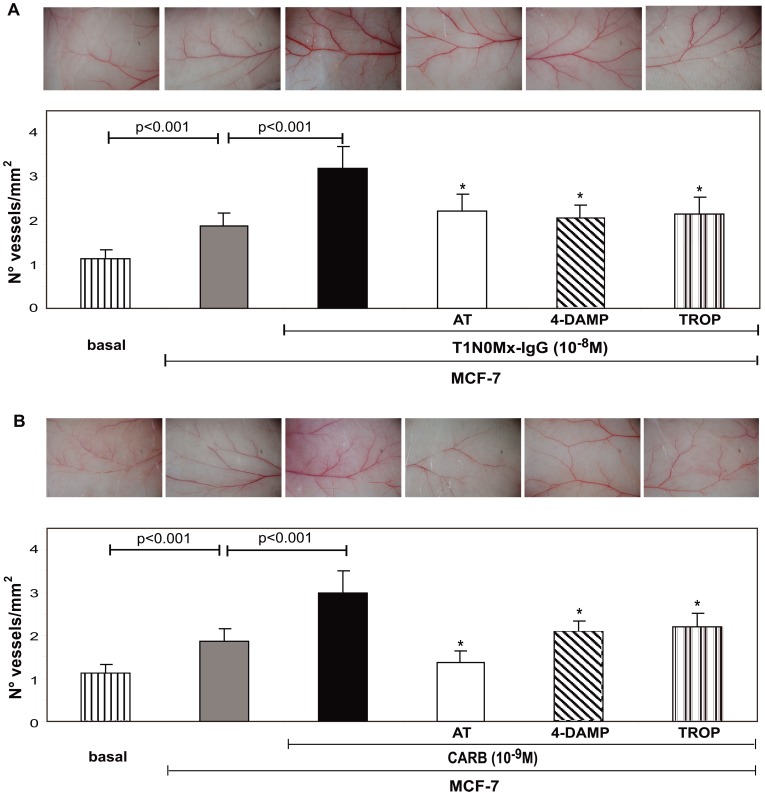
Effect of T1N0Mx-IgG or carbachol on MCF-7 cells-induced angiogenesis. MCF-7 cells were adjusted to 2×10^6^ cells/ml and treated during 1 h with A) T1N0Mx-IgG (10^−8^ M) or B) carbachol (CARB) (10^−9^ M) in the absence or presence of 10^−8^ M atropine (AT), 4-diphenylacetoxi-N-methylpiperidine (4-DAMP), or tropicamide (TROP). Cell suspensions (0.1 ml) were inoculated (i.d.) in both flanks of NUDE mice. Values are means±S.E. of three experiments performed in triplicate. Basal: animals without treatment. *p*<0.001 *vs.*T1N0Mx-IgG or CARB. Photographs of the angiogenic sites from each experimental group stated in A) and B) are shown in upper panels. Magnification 6.4×.

To complete these previous sets of experiments, we analyzed the effect of T1N0Mx-IgG on the neovascular response induced by MCF-7 cells in NUDE mice. We observed that human tumor cells induced a potent angiogenic response (δ = 1.87±0.3) that was significantly different from basal (untreated skin δ = 1.12±0.2) (Fig. 5A). T1N0Mx-IgG, obtained from five different patients increase tumor neovascularization (δ = 3.18±0.45) and we proved that this effect was due to mAChR participation since the preincubation of MCF-7 cells with atropine significantly reduced this action (δ = 2.21±0.3 *vs.* MCF-7 cells without treatment; *p*<0.1) (Fig. 5A). Photographs of the angiogenic site for each experimental group are shown (Fig. 5A upper panels). The addition of 10^−8^ M normal-IgG or BFA-IgG did not modify the angiogenic response induced by MCF-7 cells (normal-IgG δ = 1.82±0.19, BFA-IgG δ = 1.67±0.21, *p*>0.05 vs MCF-7 cells without treatment, n = 3). We confirmed that carbachol (10^−9^ M) activates mAChR expressed in tumor cells and increased the neovascular response induced by them (59±18%). The preincubation of tumor cells with atropine reduced angiogenesis to basal values (Fig. 5B). MCF-10A cells were unable to modify vessel density in NUDE mice skin (δ = 1.46±0.39, *p*>0.05 in comparison to control, untreated cells). Photographs of the vascular pattern exerted by cells treated with carbachol in the skin of NUDE mice for each experimental group are also shown (Fig. 5B).

**Figure 6:**
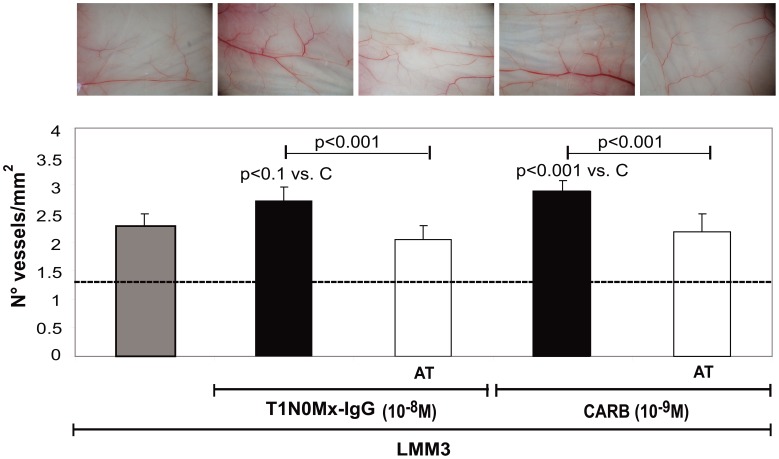
Effect of T1N0Mx-IgG or carbachol on LMM3 cells-induced angiogenesis. Cells were adjusted to 2×10^6^ cells/ml and treated during 1 h with T1N0Mx-IgG (10^−8^ M) or carbachol (CARB) (10^−9^ M) in the absence or presence of 10^−8^ M atropine (AT). Cell suspensions (0.1 ml) were inoculated (i.d.) in both flanks of BALB/c mice. Values are means±S.E. of three experiments performed in triplicate. Basal: animals without treatment. Photographs of the angiogenic sites from each experimental group are shown in upper panels. Magnification 6.4×.

In addition, we confirmed that T1N0Mx-IgG from breast cancer patients was also able to increased neovascular response induced by LMM3 cells in BALB/c mice by activating mAChR (Fig. 6). Both T1N0Mx-IgG (10^−8^ M) and carbachol (10^−9^ M) stimulated by 25±2% and 33±3% LMM3-induced vessels density in murine skin, respectively (Fig. 6 lower panel). The preincubation of cells with atropine significantly reduced IgG and carbachol action on LMM3- induced angiogenesis (*p*<0.001 vs. T1N0Mx-IgG or carbachol). Photographs of the vascular pattern in the skin of BALB/c mice exerted by LMM3 cells treated with T1N0Mx-IgG or carbachol, for each experimental group are also shown (Fig. 6 upper panel).

## Discussion

Angiogenesis, the process that leads to tumor vascularization by new blood vessel formation, is essential for tumor growth and metastasis. It is a multistep and highly regulated process in which pro-angiogenic and anti-angiogenic factors are involved [7]. VEGF is the most extensively studied angiogenic factor. The mammalian VEGF family consists of five glycoproteins referred to as VEGF-A, VEGF-B, VEGF-C, VEGF-D (also known as FIGF) and placental growth factor (PlGF) also known as PGF [15]. The best characterized of the VEGF family members is VEGF-A (commonly referred to as VEGF), is a homodimeric protein of nearly 45 kDa. It is expressed as various isoforms owing to alternative splicing that leads to mature 121, 165, 189 and 206 amino-acid proteins, which differ in their molecular weights as well as in their biological functions. The 121 isoform can diffuse and is found free in different tissues, while 165, 189 and 206 are associated to heparin with different strength. VEGF_165_ is the predominant isoform and is commonly overexpressed in a variety of human solid tumors [16]. As we observed in MCF-7 cells, soluble and membrane isoforms of this factor are both present in tumor cells, since we detected specific immunostaning either in the supernatants or in cell lysates. It is also important to note that normal MCF-10A cells did not show immunolabelling for VEGF-A, and could not stimulate angiogenic response in NUDE mice, these observations could be related with their inability to generate malignant tumors in vivo.

Previous results from our laboratory evidenced that three distinct cell lines LM2, LM3 and LMM3 originated from different spontaneous mammary adenocarcinomas arising in BALB/c mice, liberate high amounts of VEGF-A to the culture supernatants, and also expressed this factor in the cell membrane [12]. In addition, we also characterized mAChR expression in these cell lines, and we observed that all of them express the five subtypes of mAChR but LMM3 cells express 50 fold more receptor binding sites, and are more metastatic than LM3 or LM2 cells [17], [18]. We also reported that the normal murine mammary NMuMG cells lack of mAChR expression, similarly to human MCF-10A cells, and here we showed that they are not VEGF-A producers [18]. Similarly, we reported that human MCF-7 tumor cells, express mAChR (subtypes 3 and 4), meanwhile human mammary normal cells MCF-10A do not [19].

There is an important parallel between cancer and the systemic autoimmune diseases, both are characterized by a diverse collection of autoAbs directed against different antigens [20]. Most of the antigens identified in human tumors are self-proteins without mutations but inappropriately expressed or over-expressed [20]. We had previously described the presence of autoAbs in the sera of mice LM3 tumor bearers [21]. These autoAbs recognize and activate mAChR over-express in tumor cells, and promote tumor growth and angiogenesis, revealing that mAChR could be acting as autoantigens [21]. Recently, we identified a similar population of autoAbs in the IgG fraction purified from the sera of breast cancer patients in stage I [6]. Up to now, these autoAbs that interact with mAChR have been detected in all female patients studied with breast cancer in T1N0Mx [6], [22]. T1N0Mx-IgGs promote MCF-7 cell proliferation in vitro mainly through the activation of M_3_ receptor subtype that triggers phospholipase C/nitric oxide synthase metabolic pathway [22]. In addition, T1N0Mx-IgGs increased MCF-7 cell migration and invasion by up-regulating metalloproteinase-9 (MMP-9) activity in tumor cell supernatants [22]. The roles of MMPs in metastasis are multifold. They allow tumor cells to invade, intravasate and extravasate. They also allow tumor cell migration in the extracellular matrix of distant site, as well as growth and angiogenesis of secondary tumors [23]. Regarding angiogenesis, here we demonstrate that the same autoAbs that increased MMP-9 activity in MCF-7 cell supernatants can trigger VEGF-A expression and tumor-induced angiogenesis. On line with these results, Hawinkels et al. [24] reported that MMP-9 mRNA in gastric cancer tissues was up-regulated and coincided with VEGF expression and microvascular density. These findings support an emerging notion that MMP-9 is a positive regulator of tumor angiogenesis. Moreover, the latter authors showed that neutrophil-derived MMP-9 was able to release the biologically active VEGF165 from the extracellular matrix of colon cancer [24].

It is important to note that the stimulation of MCF-7 cells-induced neovascular response triggered by T1N0Mx-IgG was not totally reduced in the presence of atropine, indicating that these autoAbs could be exerting pro-angiogenic actions independently of mAChR activation, and thus deepening the effects that favor tumor growth.

Our results demonstrate that T1N0Mx-IgG were also able to up-regulate VEGF-A expression and angiogenesis induced by LMM3 cells in a more potent manner than in MCF-7 cells via muscarinic activation. This could be due the differences in the subtypes and amounts of mAChR express in both types of tumor cells as we reported previously: LMM3 cells express high amounts of the five subtypes of mAChR while MCF-7 cells only express M_3_ and M_4_ receptor subtypes [18], [19].

In conclusion our results indicate that T1N0Mx-IgG from breast cancer patients in stage I potentiate VEGF-A production and neovascular response induced by MCF-7 tumor cells by activating mAChR express in them. These autoAbs could be considered as risk factors in breast cancer progression.
